# Supplementary material for: Chromothripsis during telomere crisis is independent of NHEJ, and consistent with a replicative origin
Source: Genome Res. 2019 May;29(5):737–49. doi: 10.1101/gr.240705.118 (PMC6499312; doi:10.1101/gr.240705.118)
Supplement: Supplemental Material [file supp_gr.240705.118_Supplemental_file_1.zip › contigs/annotated_contigs/DB115/contig.2.DB115_length_406_mean_cov_5.54187192118.docx]

**DB115_length_406_mean_cov_5.54187192118**

```
TTCCAATACTGGCCACGTATTCCAAAGCACACACCAGGCAGAAAGTTACTTAGATATTCAAATCACCTCCACTCCCAGTTTACTTCATA
 >chr18:37746711-37746821 + E=1e-54
TTCGCCCCCAGCCTTCTCT|GA|CTTCAAATGTGATTCATCCATTGTGTGACACTGCTTCTCAAATTGGTGATTAACTGCCTATGTGAT
                    >chr18:37748229-37748527 + E=4e-168
CACCTGCAAACAAAAGGTTTGAAGGTATCAATTCTAGTTTTAGTTCTGCTGCTACCTTTGGTCTTGTTTTCTAATATTGTGATCCTCAA

GATCCTTCATTAAAAATATGCACAGTGATATTCTCTTCCATTTATTCCTGTTGTCCTATTTACCATAACTAATGGGGGTGATAACTATT

TACTTTTGCTGTGGACTGAATGTATATGTTCTTACTAAATTCATATGTTGAG
```
